# Cases from the Note Book

**Published:** 1859-03

**Authors:** J. R. Cushing

**Affiliations:** South Butler, Alabama


### ARTICLE II.

*Cases from the Note Book of* J. R. CUSHING, M. D. of South Butler, Alabama.

MESSRS. EDITORS—Being a diligent reader of your able and valuable Journal, especially its practical part, (to which I here acknowledge many obligations) allow a country practitioner space for a few cases from his Note Book. I have thought that many of my professional brethren were pretty much like myself, in regard to medical literature; preferring the stubborn facts of our science, *cases and their treatment.* Theory does well enough, and all that, a broad field for speculation and contrariety of opinion, where intellect can clash for mastery, or propagandists declaim it from their desks to admiring classes, all of which may be pleasant to read or hear, but let us confine it to books or controversial pamphlets; let our periodicals have more of the observations of experience, cases and their treatment, which *en passent* is a very commendable and characteristic feature of your excellent journal, "utilitarian" in every sense of the word.

To the younger members of our profession, this portion of your journal is always interesting, and studied with care, they give tone and confidence to their own timidity and inexperience—with them we cannot be too minute in the outline and therapy of a case.

Thinking a few cases from my Book may prove interesting to this class of your subscribers, I herewith forward them to you; they are taken at random from my Note Book, and may only excite interest by the novelty of the treatment:

*Case 1st.*—J. B. C——, nervo-sanguineous temperament, whom I found laboring under a severe attack of Erysipelas, seated principally on the abdomen and thorax, progressing rapidly towards the neck and head: he had taken Bitart. Potassa in small doses, also an Emetic of Ipecac without any amelioration. I found his pulse small and quick—he complained of a most intense burning over his whole abdomen and stomach, which he described as "burning, stinging and throbbing;" his tongue was heavily coated with a viscid yellow fur. Venesection in this case I considered of doubtful efficacy, the pulse not warranting depletion. I prescribed the following purgative: Pil, Hyd. gr. vi; quin. sulph. grs. x; morph. sulph. gr. ¼; M. Tt., pil, No. ii, and given immediately, which I repeated in four hours. To relieve the intolerable burning, I exhausted every remedy I could think of or ever read of; lard, ungt hyd., flour, chalk, meal, etc., without any relief whatever; poultices were applied of every description, lime liniment, and every thing else, without the least relief. After twelve hours of intense suffering, Dr. Hicks Cook, a neighboring physician, was called in consultation; at his suggestion, the mercurial not having thoroughly operated, the following formula was given: Co. ex. colocynth, gr. vi; pulv. rhu. gr. vi, which in a few hours, operated well, yet still without any abatement of the intense *burning.*

As a "dernier resort," we concluded to envelope the parts in flannel, wrung out of boiling water; it acted like a charm; giving instantaneous relief, these applications were continued consecutively every ten minutes for twenty-four hours, before the disease succumbed, being the first and only thing that gave *ease* during the whole treatment. It was the opinion of Dr. Cook, as well as myself, that there would be a general peeling off of the cuticle, but strange to say, it was not the case.

What was remarkable in this case was the absence of that peculiar thickness of the skin, this disease is so apt to be accompanied with; also the skin did not vesicate and desquamate as in usual attacks, and yet there is no doubt but it was a pure case of inflammation of the skin, and of the most violent kind at that; its progress was rapid towards the neck, being only restricted by a broad line of the tinct. of iodine, drawn around the upper part of the thorax, which being stopped in this direction, it took its course downward and terminated at the toes.

The proximate cause of this attack was, no doubt, portal and biliary engorgement; the disease itself terminating in a Diarrhœa of a very dark and offensive billious matter.

*Case 2d.*—Mrs. P—— called upon me to "regulate her," as she termed it, age 22 years; had been married five years, and had had no children. From her statement, she appeared to be laboring under Dysmenorrhœa; her general condition was anemic; had been in bad health ever since her marriage, and for some time before. From the diagnosis, I put her upon the following plan of treatment: Subcarb. ferri, ℥i; pulv. gentian, ℥i; pulv. columbo, ℥i; pulv. ginger, ʒiv, mixed thoroughly; dose, teaspoonfull three times daily, in a little water, with the following vaginal wash: Act. plumbi. ʒii; sulp. zinci, ʒi; aqua pura, Oi, three times a day.

One month from the above prescription, I called upon her, and found but little improvement in her monthly sufferings; she also complained of an extensive pruritus that occasioned her a great deal of suffering. I prescribed dusting with hyd. submu. according to Dr. Dewees' formula, and also gave her his preparation of vol. tinct. guiac, teaspoon doses three times a day, with a tart. emt. plaster above the symphisis pubis.

Nov. 20th. Eighteen days after the above, I was visited by my patient—I have neglected to say, that I had previously examined her womb per speculum, which I found in an apparently healthy condition; the only abnormal feature I discovered being the extreme pruritus of the external parts, to which I attributed the pain she complained of during conjugal embraces. She now complained of an extreme flow of "whites," which she informed me commenced during her last catamenial discharge, and had continued ever since, with an aggravation of the "pruritus."

Nov. 22d. Visited patient; on examination, per speculum, I could discover no ulceration or visible inflammation of the os; from this, was I led to believe, that the whole mischief was within the neck of the uterus itself. After a good deal of manipulation, I introduced a large catheter and produced a sufficient dilatation; I used the Solid nit. argenti, ʒss, to ounce of aqua rosa, with a female syringe, through the speculum, pressed firmly against the os, after which, using Ulmus Fulva washes several times a day. This I continued once or twice a week for two months; during the interim, giving her Fowler's sol. dose v to x gtt. twice daily. In February I discharged her, "regulated" *enciente.*

In the November following, one year, from my first seeing her, I was called to wait upon her during parturition, and after twenty-two hours tedious labor, delivered her of a fine healthy boy, weighing 11¾ pounds. Comment on this case is unnecessary; the point to be noticed is, that many of the catamenial irregularities are owing to the diseased state of the internal surfaces of the neck of the uterus, without any apparent lesion of the os itself. Since this case I have had several cases of amenorrhœa, as well as dysmenorrhœa, and have been invariably particular, in exploring the womb for the latent cause, when they resisted ordinary remedies.

